# Rationale of an Advanced Integrative Approach Applied to Autism Spectrum Disorder: Review, Discussion and Proposal

**DOI:** 10.3390/jpm11060514

**Published:** 2021-06-04

**Authors:** María Luján Ferreira, Nicolás Loyacono

**Affiliations:** 1 TEA-Enfoque Integrador Group, Bahía Blanca 8000, Argentina; ferreiramaralujn@yahoo.com; 2 SANyTA (Sociedad Argentina de Neurodesarrollo y Trastornos Asociados), Migueletes 681, Piso 2, Departamento 2, BUE-Ciudad Autónoma de Buenos Aires C1426, Argentina

**Keywords:** integrative, model, ASD, concomitant, condition, disorder

## Abstract

The rationale of an Advanced Integrative Model and an Advanced Integrative Approach is presented. In the context of Allopathic Medicine, this model introduces the evaluation, clinical exploration, diagnosis, and treatment of concomitant medical problems to the diagnosis of Autism Spectrum Disorder. These may be outside or inside the brain. The concepts of static or chronic, dynamic encephalopathy and condition for Autism Spectrum Disorder are defined in this model, which looks at the response to the treatments of concomitant medical problemsto the diagnosis of Autism Spectrum Disorder. (1) Background: Antecedents and rationale of an Advanced Integrative Model and of an Advanced Integrative Approach are presented; (2) Methods: Concomitant medical problems to the diagnosis of Autism Spectrum Disorder and a discussion of the known responses of their treatments are presented; (3) Results: Groups in Autism are defined and explained, related to the responses of the treatments of the concomitant medical problems to ASD and (4) Conclusions: The analysis in the framework of an Advanced Integrative Model of three groups including the concepts of static encephalopathy; chronic, dynamic encephalopathy and condition for Autism Spectrum Disorder explains findings in the field, previously not understood.

## 1. Introduction—The Background

This manuscript presents the rationale of a new model of approach to Autism Spectrum Disorder. There are several acronyms that will be used throughout this work:

Autism Spectrum Disorder = ASD
Advanced Integrative Model = AIM, a new model of approach to ASD
Advanced Integrative Approach = AIA. The AIA is the application of the AIM.
Concomitant medical problems to diagnosis = CMPD. The CMPD are the medical problems outside the brain (mainly competence of the General Practitioner in adulthood and Pediatricians in childhood) and inside the brain (competence of the fields of Neurology and Psychiatry).
Neuro-Developmental Disorders = NDDs
Diagnostic and Statistical Manual of Mental Disorders, fourth version = DSM-IV
Diagnostic and Statistical Manual of Mental Disorders, fifth version = DSM-5
Genetic Model = GM
Intellectual disability = ID
Attention deficit hyperactivity disorder = ADHD
Obsessive-compulsive disorder = OCD

### 1.1. One Finding, One Treatment—The Old Era of Simplification as the Goal

Most recent manuscripts introduce Autism Spectrum Disorder or ASD as “Autism spectrum disorder defines a broad group of NDDs characterized by (i) young age of onset, (ii) impairment in communication and social abilities, (iii) restricted interests and repetitive behaviors, and (iv) symptoms that affect patients’ function in various areas of their life”. Many of today’s manuscripts about Autism Spectrum Disorder (ASD) begin with the phrase: “The complex pathophysiology of autism spectrum disorder encompasses interactions between genetic and environmental factors” or similar [1]. The diagnosis of ASD is given considering the Diagnostic and Statistical Manual of Mental Disorders (DSM) in 2021, with its fifth version or DSM-5. A recent review summarized the history of the DSM from Kanner to DSM-5 [2]. In precision medicine, the gap between bodily behaviors and genomics is being addressed, including the study of gene expression on tissues beyond the brain, in organs for vital functions. This approach is proposed to reframe Psychiatry [3].

Recent manuscripts reviewed the so-called comorbidities in ASD [4,5]. Comorbidities may be psychiatric [6], neurological [7], or related to medical conditions beyond the brain in the field of Pediatrics or General Medicine [8]. Recent literature has demonstrated that people with ASD diagnosis may have multiple comorbidities in different combinations and severity [9] and even temporal, transient hyper-multimorbidity. Multimorbidity is present when multiple medical issues (called comorbidities) are diagnosed in the same person. The present manuscript will call the medical issues that are frequently present in people with ASD as “concomitant medical problems to diagnosis” (of ASD) or CMPD. These CMPD are outside the brain or related to the brain (neurological and psychiatric CMPD of ASD). Previous attempts proposed potentially different roles for CMPD [10].

### 1.2. Trans-Discipline for the Analysis of the So Called “Comorbidities”

Complexity science forces us to see the dynamic properties of systems and the varying properties that are related to social roots [11,12]. ASD may be considered a complex diagnosis that resists the finding of new approaches via traditional models. It would be better tackled through interdisciplinary, systems-level approaches, considering implementation science [13,14].

Somatic health is a key point to move forward [15]. Several important reports have alerted about the need for the serious consideration of the CMPD of ASD [3,4,16,17,18] with transdisciplinary and interdisciplinary collaboration in the context of the multimorbidity [19]. As reference [17] cites, ASD is defined behaviorally. It includes the consideration of impairments in social behavior, stereotypic movements, and communication issues with impact on social skills, called “core symptoms of ASD”. All these symptoms significantly impair the quality of life of people diagnosed with ASD [20,21]. 

Medical conditions such as gut dysbiosis [22], non-celiac gluten sensitivity [23], cerebral folate deficiency [24], food allergies and intolerances [25], gastrointestinal [26], metabolic [27] and biochemical issues [28], immune dysfunction [29], autoimmune problems [30], mitochondrial dysfunction [31], barrier permeabilities [32], oxidative stress [33], endocrine issues [34] and more are not explored (sometimes for years) in ASD patients. The most advanced approaches have shifted the focus of the “causation search” original framework to the study of the (epi) genetic susceptibility for ASD, from the brain to the whole body [1,35], and to the importance of humanism in medicine [36] as well as to the study of genes expressed in tissues outside of the brain [37]. As Constantino recently reported, the so-called “co-morbidities” of ASD are inappropriately named if they actually contribute to (or exacerbate) the severity of autism itself [38]. Multimorbidity affects the generation of evidence [39] and a new Evidence Pyramid in Evidence Based Medicine was recently proposed [40]. Multimorbidity and hyper-multimorbidity should be taken into account in the case of ASD. The field of ASD needs personalized medicine as the norm.

### 1.3. What This Manuscript Is

This manuscript is not a narrative or scoping review in a traditional sense [41]. It is not a systematic review, meta-analysis or meta-synthesis. Furthermore, it does not propose a medical hypothesis.

This manuscript presents a New Model, the Advanced Integrative Model (AIM) and its application, the Advanced Integrative Approach (AIA). AIA is the application of the AIM. Evaluation, diagnosis, and treatment of CMP to ASD diagnosis are very important in this model, considering those in the brain and beyond the brain. Mainly reviews and systematic reviews in CMPD were included in the revision. The selected language was English. These manuscripts were selected using PubMed, Scopus, and Medline, with keywords such as “ASD” and “health comorbidities”, “health children”, “physical health teen-adolescents”, “physical health adults”, “quality of life”, “outcomes”, “gastrointestinal”, “immune”, “autoimmune”, “mitochondria”, “symptoms”, “physical conditions” and combinations of them with systematic review/review or general articles. Published manuscripts about prevalence, advocacy, neurodiversity, and genetics in ASD were also included. The publication dates of the 73 references are from 2011 to 2021, with nearly 24 published in 2020–2021 and only 4 before 2010.

The design and answer of three main questions relating to a person (child/teen/adult) diagnosed with ASD (but presented for a child) are discussed in this manuscript. These main questions are:

*What does ASD mean?*
*How CMPD can be evaluated, diagnosed and treated rigorously and adequately today in this child with ASD?*
*Which would be the best combination of medical and non-medical tools for this child, considering the whole-body status at this moment in the Advanced Integrative Approach (AIA) thinking in multimorbidity?*

## 2. When the Conclusion Should Not Be the Presumption

Looking at the published research in ASD, there is plenty of information about neurological [42], psychiatric [43] and biological (outside the brain) CMPD of ASD [3]. These are almost always called comorbidities. However, comorbidities mean that medical conditions present are not related to a main diagnosis, in this case ASD. The design of the research studies in ASD is performed considering OFAT (one factor at a time) instead of the context of multimorbidity. ASD is a model psychiatric disorder following the DSM-5 for the analysis of multimorbidity and personalized medicine.

In this case, multimorbidity is present in the brain (psychiatric and neurological issues) and outside the brain (biological problems in body systems outside the brain) with behavioral, emotional, motor, sensorial and communicational symptoms. The physicians related to these areas are from Psychiatry, Neurology and Pediatrics. The Pediatrician detects ASD and refers to other areas. However, in the Advanced Integrative Model the Pediatrician (or General Practitioner) by training, experience, and competence, is of paramount importance in the Advanced Integrative Approach.

### 2.1. The Response to Treatment of CMPD in ASD

When a family receives a diagnosis, CMPD are considered comorbid, not related to ASD and of little or no impact on core symptoms or trajectory of ASD. The recommended practice, if it includes exploration of CMPD, is only the limited exploration of gastrointestinal issues, beyond the neurological or psychiatric co-occurring medical problems. In a recent manuscript the recommendations were educational practices, developmental therapies, and behavioral interventions, but CMPD (in particular the out-of-the-brain biological issues) were not properly considered in the state-of-the art knowledge [44]. It has not been considered, historically, that the results of all the behavioral, relational, developmental or psychoeducative methods to approach ASD are strongly related to the biological status of the person with the ASD diagnosis.

The question is how could the treatment(s) of CMPD affect the ASD symptoms?

There are several possible outcomes to the treatments of CMPD of ASD outside the brain.

There are people diagnosed with ASD (mainly children) whose core ASD symptoms disappear after the adequate treatment of CMPD outside the brain. ASD symptoms seem to be only symptoms of a few CMPD outside the brain with a causal relation [45].

There are people diagnosed with ASD (all ages) whose core ASD symptoms ameliorate after the adequate treatment of CMPD outside the brain. Many times, several CMPD need to be considered and properly treated to show an impact in the core symptoms. ASD symptoms appear to be related to CMPD in ASD [46].

There are people diagnosed with ASD (all ages) whose core ASD symptoms do not change after the adequate treatment of CMPD outside the brain, even when several CMPD are considered and properly treated. ASD symptoms are not related to CMPD in ASD. In this case, they could be called “comorbid”.

The individual response to the most rigorous, controlled, and serious allopathic treatments of CMPD in ASD, taking into account multimorbidity and complexity, gives clues to their roles. Therefore, the role of CMPD in ASD would be shown or concluded after and not before the treatment of them.

As Dr. Frye’s group has reported, in ASD many neurological issues have links to biological problems not related to the brain [47]. Dr. Frye has published several important manuscripts about CMPD in ASD and from the design the work is presented differently than other manuscripts. The titles of these manuscripts generally are “XXX as treatment of YYY in ASD”. This kind of approach to the problem takes into account, since the design, the multiple CMPD in ASD. Historically the presentation of the treatment of CMPD was “XXX as treatment of ASD”. Many recent manuscripts detect, count, and report the so called “comorbidities” instead of considering new models for the role of these CMPD [4,48,49]. Not all children with gut dysbiosis have ASD, not all children with mitochondrial dysfunction or with some immune deficiency have ASD. There should be another component to take into account and address this complexity and this other component is the brain status in ASD.

### 2.2. The Controversy about Whether a Static or Dynamic Encephalopathy Contributes to ASD

Encephalopathy is a term used here for a diffuse disorder (or disease) that alters brain function or structure. An encephalopathy is dynamic when it responds to treatments of CMPD outside the brain. An encephalopathy is static when it does not change; it does not respond to treatments of CMPD. A central point is if the encephalopathy in ASD is static or dynamic and how. The dynamic encephalopathy in ASD is considered at first to be chronic and difficult to change, once present. The dynamism of the encephalopathy would also be related to the plastic nature of the brain and the number, combination, and severity of the CMPD. The development of the encephalopathy and the path to chronicity of it is then considered a process, not a genes-mediated fact for all people diagnosed with ASD. This process may begin prenatally (as vulnerability and/or through a genetic mutation/s or polymorphism/s and combinations of them with environmental impact) and/or postnatally. The mechanisms for the encephalopathy to develop involve the genetic susceptibility to CMPD in the brain and outside the brain and the individual response to in-series and in-parallel exposures in the second decade of the XXI century. From processed food to antibiotics, from contaminated water and air to mitochondrial impact, from dysbiosis to whole-body dysfunction and more the many pathways to gut barrier permeability and brain–blood barrier permeability in vulnerable people are explained looking at the model of ASD as symptoms of a dynamic (but chronic) encephalopathy.

Since the presentation of the genetic model (GM) with the manuscript of Folstein and Rutter [50], 44 years has shown the exploration of the genetic basis of ASD. Meanwhile, the prevalence has grown up to 1 in 54 from the CDC data [51], that is more related to 1 in 36 [52] and near 1 in 20 males in children up to 17 years or even higher [53]. The main point of the GM is the consideration of the root of ASD as a static encephalopathy of prenatal origin [54]. In the neurodiversity model, ASD is a way of being [55]. These two points of view do not explain many findings, do not give tools or resources to professionals, non-professionals and families to address the individual complex medical, non-medical, and educational needs of many children, teens and adults diagnosed with ASD. There are several recent reports about these unmet needs [56,57,58].

The Advanced Integrative Model (AIM) is a new model of ASD. In this model the CMPD outside the brain should be properly diagnosed and treated. These CMPD may be related to a chronic encephalopathy through the barrier’s permeability. Gut and brain blood barrier permeabilities are important to understand in this proposal. The gut dysbiosis involves pathogenic bacteria, parasites, and fungus that may translocate and/or produce metabolites and correlates with inflammation in the presence of a permeable gut barrier. This abnormal situation produces an immune response. The immune system components and metabolites from gut dysbiosis reach the bloodstream due to the permeable gut barrier and finally the brain due to brain blood barrier permeability [15]. The idea of a chronic, dynamic encephalopathy as a model of ASD was presented by Dr. Herbert in 2005 [59] but unfortunately was not explored adequately up until the last 10 years and much more in the last 5 years.

In the framework of a model based on the explanation of ASD as a static encephalopathy of prenatal origin, the plausibility of a role of postnatal development disturbance is not taken into account and dismissed. CMPD have been labeled as “comorbid”: medical issues that have no link to ASD. Coincidence or simply better health has been the explanation for the reported improvements after treatments of CMPD, which are sometimes very dramatic. Regression (loss of speech and/or abilities and/or skills) continues to happen today without explanation in these models. No other proposals have been presented, even when no genetic link in brain to regression can be clearly shown in ASD [60]. Today, regression has been reported to be present in prospective studies in up to 88% of people with ASD [61], although the consensus in retrospective studies is lower and nearer 30% [62]. Regression is understood in AIM as the final point of a pre-encephalopathy process. The pathway to chronicity is considered to be an individual process and not a single event [63] and the final point could be considered to be the regression. The chronic status of the encephalopathy would be related to chronic pathophysiological processes in the brain in ASD (see reference [52] for further explanation).

## 3. AIM Classification System

Core symptoms of ASD include impairments in social interaction and communication, and restricted and repetitive behaviors. There are no known efficacious treatments for the core social symptoms, although effects on repetitive behaviors have been reported [64].

The main groups in ASD following the AIM would now be:

Main Group 1—Core ASD symptoms disappear after the adequate treatment of CMPD outside the brain. In this case the encephalopathy is dynamic and completely reversible with loss of the ASD diagnosis.

Main Group 2—Core ASD symptoms ameliorate after the adequate treatment of CMPD outside the brain. In this case the encephalopathy is dynamic but chronic, partially reversible, with improvements in ASD symptoms from mild to huge, even without loss of the ASD diagnosis.

Main Group 3—Core ASD symptoms do not change after the adequate treatment of CMPD outside the brain, even when several CMPD are considered and properly treated. Some people diagnosed with ASD without intellectual disability (ID) and Asperger’s syndrome following DSM-IV would be a subgroup where ASD is related to a condition as a way of being. Other subgroups would have strong links to genetics, with ID besides the ASD diagnosis, and the ASD symptoms would be related to a static encephalopathy in the subgroup called “syndromic autism” [65]. These subgroups are very different.

**Figure 1:**
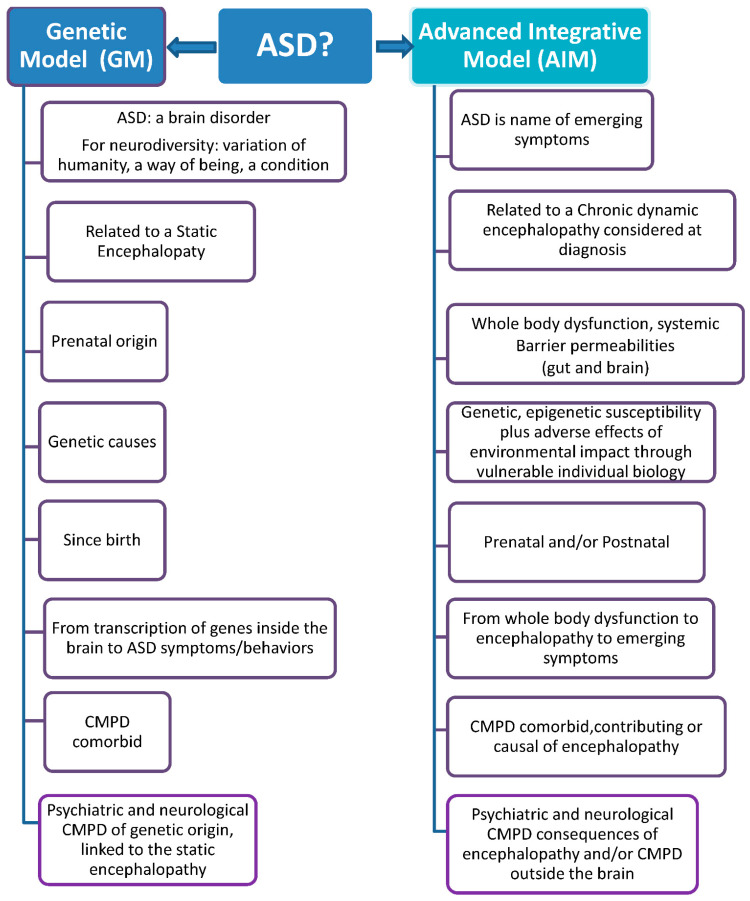
Genetic Model versus Advanced Integrative Model.

In these three main groups, many different subgroups may be defined, considering ID or not, speech problems, sex, age and more. Figure 1 shows the comparison between the Genetic Model (GM) and the Advanced Integrative Model (AIM) in their answer to the first of the three important questions this manuscript presents: What does ASD mean?

## 4. The Advanced Integrative Approach (AIA)

The second key question is what causes ASD? For the GM, the answer is genes or genes plus environment at the prenatal step or genes and epigenetics plus environment at the prenatal step. The manuscripts dealing with an important number of CMP generally obtain the information from medical records and report statistics. It is known that medical records in ASD are very incomplete because, many times, the extensive biological exploration outside the brain in ASD does not exist. The experience and the research show that many times, a person (child, teen or adult) with ASD diagnosis have CMPD in ASD outside the brain and neurological (seizures/epilepsy, movement disorders, sleep disorders), psychiatric diagnosis (from attention deficit hyperactivity disorder or ADHD to bipolar disorder, from anxiety to OCD/tics and more), language/speech problems (all the spectra of them), learning challenges, a spectra of intellectual disability and behavioral, emotional and psychological issues.

The question “What causes ASD in all children?” has shifted following the last 10 years of research to “What causes ASD in this child?”

The Advanced Integrative Approach (AIA) is the AIM in practice. The AIA is personalized and the answer to the question presented above is not straightforward. The model gives tools and resources to help and explore in different ways if medical problems outside the brain and at systemic level are at least part of the answer to that question for the person diagnosed with ASD.

Therefore, in an AIA, the second question is How CMPD can be evaluated, diagnosed and treated rigorously and adequately today in this child with ASD?

Medical and non-medical professionals do not receive the information about CMPD in ASD. If they receive it, it is incomplete or it is presented as hypothetical or “alternative” (when it is not). Discussion about models has been focused on neurodiversity versus the so called “medical model”. This “medical model” includes the treatment of ASD in the context of the GM (with behavioral approaches, psychoeducative methods, and psychopharmacology). The individual symptoms of a person with an ASD diagnosis give the trained physician clues about what to explore. Many times symptoms of CMPD in ASD are only behavioral, emotional, or related to aggression/auto-aggression and more [66]. Disruptive behavior should be analyzed first, as a request for help due to potential pain, discomfort or an altered brain state [67].

**Figure 2:**
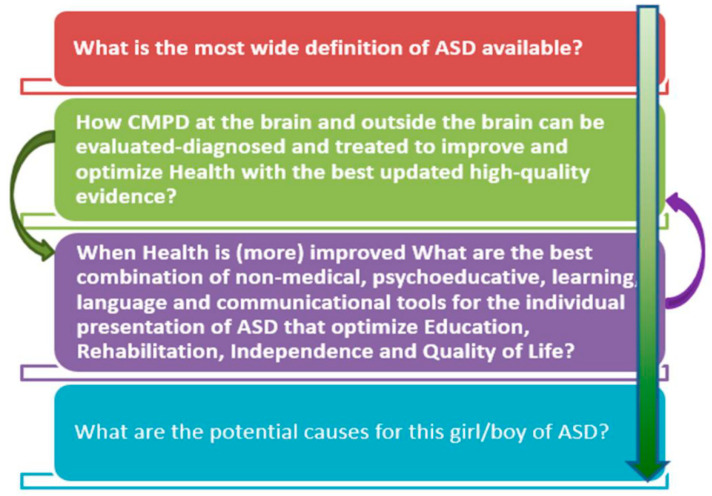
Main questions to present and to take into account.

The third question would be which would be the best combination of medical and non-medical tools for this child, considering the whole-body status at this moment in the Advanced Integrative Approach (AIA) thinking in multimorbidity? Figure 2 shows the hierarchy of the questions discussed in this section, expanded. The arrow shows the increasing difficulty of the questions to be answered. The last question is the most difficult to answer.

## 5. When Genetics Is an Important Part in Building Individual Vulnerability

The genetics in ASD is of paramount importance. Genetic susceptibility in ASD is being presented more and more as increased vulnerability for the CMPD to develop. This analysis gives support to the genetics and epigenetic links in ASD but at a whole-body level, not only the brain [2].

Here, we present a new model called AIM that aims to represent a more comprehensive model for ASD:

From Genetic Susceptibility to Mutated Genes-Whole Body (including brain)-CMPD (in and outside the brain)-Permeable barriers-Chronic, Dynamic, Systemic (to Static) Encephalopathy or Condition-ASD Behavior.

In the AIM the role of genetics is different but no less important than in the GM.

## 6. Beyond the Brain and the System’s Biology Updated to 2021

For many years, there has been a long discussion about if what is needed is health or education in the field of ASD. From the application of the AIM to an AIA, the answer is one or the other or both, depending on the individual ASD age, sex and individual presentation.

Main groups one to three include people diagnosed with ASD of all ages and presentations whose core ASD symptoms respond (or not) to treatment of CMPD, including people with Asperger’s syndrome (following the DSM-IV), today included in ASD (following DSM-5). The needs are different in each main group in terms of education and/or health and other services. When core symptoms of ASD respond to treatment of CMPD, they are not a way of being or a condition, they are emerging symptoms. When core symptoms of ASD do not respond to the treatment of all the CMPD when properly and exhaustively considered, then the diagnosis of ASD could be related to a way of being for that particular person or to a static encephalopathy.

With adequate training, many symptoms historically assigned to “autism” may guide the clinician to the diagnosis and treatment of CMPD in ASD [3,4]. The treatments of CMPD are only that, they are not “ASD treatments”. Even if core ASD symptoms do not respond, the treatment of adequately diagnosed CMPD in the main group three would improve the quality of life. The AIM involves the application in practice of the system´s biology updated to 2021, with the consideration of ASD as a whole-body disorder.

### 6.1. Why Is the Progress So Slow in Practice When So Much Is Known from Published Research?

Published research has been increasing regarding CMPD in ASD. The main groups one and two have not been properly studied from CMPD point of view. One of the most cited problems has been the confounding role of the intellectual disability (ID). This group also has increased the number of CMPD in ASD [3,8]. A selection bias to study people with ASD without ID was shown [68,69].

Even when it is clear that there are different subgroups of people diagnosed with ASD that respond positively, and sometimes spectacularly, to the treatment of CMPD; even when the information about these subgroups are data, not anecdotal; even when the optimal outcome was reported by several groups around the world [70,71]; even when the individual improvements after proper treatments were reported in many properly presented reports [24,39,72]; even when there are several manuscripts that show the importance of the individual response to CMPD [2], the research funding in CMPD has not been enough and in general only 9% was assigned to services research in ASD [73]. The updated information is not reaching the local sources of trusted information for doctors in practice.

The response to the treatment of CMPD gives clues to the nature of the underlying encephalopathy: static o dynamic, reversible or irreversible. The AIM allows and explains the possibility of a partial reversibility of the encephalopathy and (less reported as yet, possible) total reversibility and loss of an ASD diagnosis.

### 6.2. Advanced Integrative Approach (AIA): A New Model Taking Science into Medical Practice with Permanent Review

**Figure 3:**
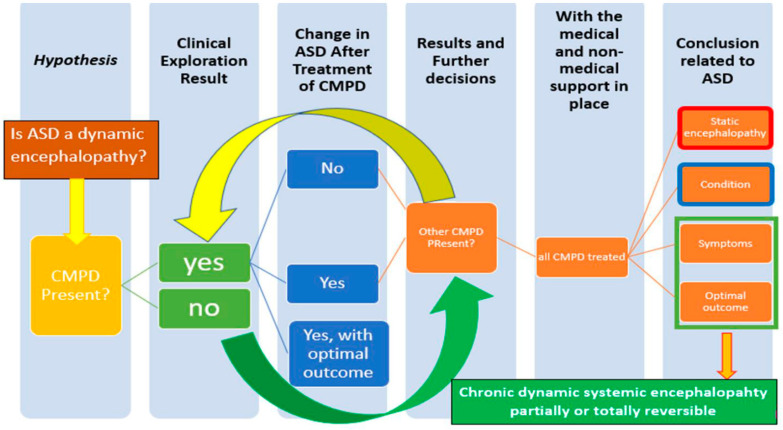
A path for the physician from hypothesis to conclusion in an Advanced Integrative Approach (AIA) of ASD.

The application of an AIM requires Implementation Science, a careful design of the optimization of translational research. The higher the severity of ASD, the higher the number, combination and complexity of the CMPD. In physician practice (Pediatrician or General Practitioner), the AIA involves the careful consideration of the CMPD in ASD from the beginning. Figure 3 shows the sequence of learning about CMPD. First, the hypothesis in the AIA is that a chronic dynamic encephalopathy is underlying with the ASD diagnosis. Later, after a careful clinical exploration (also involving professionals from Neurology, Psychiatry and Genetics, if needed), CMPD are diagnosed and treated, sequentially or in parallel as needed. Finally, with the response to treatments of the CMPD in ASD the conclusion for the individual with ASD can be obtained: static encephalopathy, dynamic encephalopathy (partially or completely reversible), or condition. The complexity of the ASD presentation, the behavioral, emotional, sensorial, motor and speech/communication symptoms, and the CMPD from Pediatrics, Neurology, and Psychiatry plus their treatments require permanent review and updates.

## 7. Conclusions

The Advanced Integrative Model (AIM) and the Advanced Integrative Approach (AIA) are presented and explained in this manuscript. The consideration of the CMPD to ASD from an individual point-of-view and the analysis of the response to the proper treatment of all of them are the key to present the three different main groups. Considering the response to treatments of the CMPD of ASD, the conclusion for the individual ASD may be related to a static encephalopathy, to a chronic dynamic encephalopathy (partially or completely reversible) or to a condition. The Advanced Integrative Model for ASD includes the GM and also the idea of ASD as a condition for a subgroup of people diagnosed with ASD.
